# Reconstruction of Secondary Type Congenital Hallux Varus with Modification of the Farmer Technique for Cover of Skin Defect: Report of Three Cases

**DOI:** 10.5704/MOJ.1911.013

**Published:** 2019-11

**Authors:** AR Sulaiman, I Munajat, H M-Yusuf, NMS Nik-Jaffar, NH Zarullail, EF Mohd, NA Johari

**Affiliations:** Department of Orthopaedics, Universiti Sains Malaysia, Kubang Kerian, Malaysia; * Department of Orthopaedics and Traumatology, Hospital Kuala Lumpur, Kuala Lumpur, Malaysia

**Keywords:** congenital, hallux varus, excess skin, extra digit, modified technique

## Abstract

The reconstruction of hallux varus deformity involves the release of contracted medial structure and realignment of the phalange, leaving a significant skin defect which requires cover. Farmer described proximal based rotational skin flap from the first web space to cover the defect. This technique may compromise the circulation to the flap and risk to the lateral digital vessels. We modified his technique to address these issues. We report a successful reconstruction using the Farmer’s technique on one patient and a modified technique on three patients. We used the excess skin from the extra digit to cover the medial defect. We found this modified technique of skin cover safe without risk of injuring the neurovascular bundle. There was no recurrence of deformity at last foolow-up. All patients were able to wear normal shoes.

## Introduction

Congenital hallux varus is a rare deformity in which the great toe is deviated medially at the metatarsophalangeal joint with an increased width of the first web space. In the secondary type or associated type of congenital hallux varus the condition is associated with other congenital deformities of the forefoot. There is presence of a firm fibrous band along the inner side of the adducted great toe causing the toe deviation. McElvenny described the correction of hallux varus by release of the medial structure and realignment of phalanges of the great toe to the head of the metatarsal, creating the medial skin defect^1^. Farmer described the creation of broad proximal based skin-fat flap from the plantar or dorsal surface of the first web space, rotated to cover the medial skin defect^2^. The alignment was maintained by creating syndactyly between the first and the second toes. However, there is a risk of vascular compromise on the rotated skin flap. We report successful results of primarily modifying the technique for cover of the medial skin defect using the excess skin from the extra digit, while the release of the internal medial structures is similar to the Farmer technique.

## Report of Three Cases

There were four patients in our series. The patients were between four and nine years of age at the time of surgery. Clinically, three patients had hallux varus with polysyndactyly and one case with polydactyly. All these patients were secondary type or associated type of congenital hallux varus in which the hallux varus were associated with other congenital deformities of the forefoot such as polysyndactyly or polydactyly like in our cases. Further details of the cases are described in Table I. One case underwent Farmer’s technique while the other three cases underwent modified technique. We considered a good outcome if the reduction was maintained with no recurrence of deformity besides the ability to wear normal shoes. There was no recurrence on the last follow-up.

**Table I:** Summary on the age at time of surgery, clinical features, types of reconstructive surgery performed, outcome and follow-up period in all four cases

Case	Age at surgery	Clinical features	Surgeries performed	Outcome / period of follow-up
1	7-month-old	1. right hallux varus	1. medial release and realignment	Good / 8 years
		2. polysyndactyly	of big toe	
		3. normal 1st metatarsal	2. Farmer’s procedure	
2	2-year-old	1. left hallux varus	1. medial release and realignment	Good / 4 years
		2. polysyndactyly	of big toe	
		3. short 1st metatarsal	2. resection and enucleation of phalanx	
			3. usage excess of plantar skin	
3	3-year-old	1. left hallux varus	1. medial release and realignment	Good / 1 year
		2. polysyndactyly	of big toe	
		3. short 1st metatarsal	2. enucleation of phalanx	
			3. usage excess of plantar skin	
4	2-year-old	1. right hallux varus	1. medial release and realignment	Good / 6 years
		2. polydactyly	of big toe	
		3. short 1st metatarsal	2. excision of phalanx	
			3. proximal based flap	

There were two types of surgical procedures in these series depending on the clinical presentations. All patients went through medial soft tissue release as described by McElvenny. The exposed reconstructed capsule over the metatarsophalangeal joint area left after the realignment of the hallux varus was closed with three different methods.

**Fig. 1:**
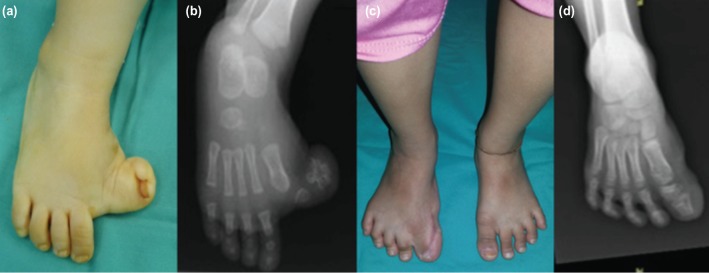
(a) Pre-operative photograph of case No. 1 shows a severe right hallux varus with wide first web space and polysyndactyly of the right big toe. (b) Radiograph shows a bifid distal phalanx of the right hallux. (c) Post-operative photograph after Farmer’s procedure with creation of syndactyly of the first web space and realignment of the right big toe. (d) Post-operative radiograph shows a well reduced first metatarsophalangeal joint and a corrected right hallux varus.

The first method was Farmer’s technique on patient No. 1 with bifid hallux varus (Fig. 1a, 1b, 1c, and 1d). In this patient, the plantar proximal base flap was elevated from first web space, rotated to cover the skin defect medially. The skin defect created from flap elevation in the first web space was closed by creating syndactyly between the right big toe to the second toe. It helps in maintaining the alignment of the big toe. In this patient, we could not maintain the big toe alignment with K- wire because the circulation of the big toe was compromised after K-wire insertion. Instead, cast with medial extension to toe was applied. The K-wire was reinserted back after two weeks when the circulation of the flap toe was stable, and removed at six weeks.

**Fig. 2:**
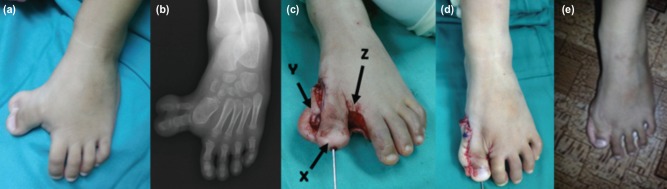
(a,b) Pre-operative photograph and radiograph of case No. 2 show polysyndactyly and congenital hallux varus of the left big toe. (c) The realigned big toe ‘x’ held with K-wire after medial soft tissue release with plantar based skin flap ‘y’ after filleting of bone in the extra digit and resection of the skin in the first web space ’z’. (d) The extra skin sutured to cover the medial skin defect and the first web space closure. (e) Post-operative photograph after 3 years.

We used a modified technique in patient No. 2 and patient No. 3 with polysyndactyly (Fig. 2a and 2b). We made the incision in between the syndactyly and performed a medial soft tissue release through it (Fig. 2c). This allowed the realignment of the hallux varus leaving the skin gap medially. Through the same wound, we filleted the phalanx of the medial extra digit. The skin of the extra digit, after removal of excess soft tissue, became a plantar based skin flap which was used to cover the defect created by realignment of the hallux varus (Fig. 2c and 2d). The excess skin in the first web space was excised and primarily closed without creating syndactyly (Fig. 2c, 2d and 2e).

**Fig. 3:**
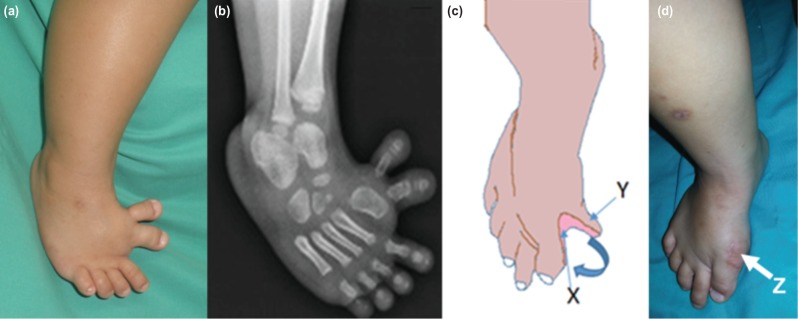
(a and b) Photograph and radiograph of case No. 4 with hallux varus and well separated polydactyly. (c) The illustration with arrow ‘x’ showing a skin defect after realignment of the big toe and arrow ‘y’ showing the proximal based skin flap created from resection of the bone and soft tissue from accessory medial toe. (d) Post-operative photograph one year after the modified reconstruction procedure with arrow ‘z’ showing a healed proximal based skin flap.

The third procedure was performed on patient No. 4 with a clear polydactyly without syndactyly (Fig. 3a and 3b). The incision was made between the hallux varus and the extra digit to release the medial structure. The base of hallux varus was accessed through the wound, and the medial cord was released. The big toe was then repositioned to be in line with the first metatarsal and transfixed with a 1.6 mm K-wire. The big toe was realigned resulting in a skin gap (Fig. 3c). The bone and the soft tissue in the extra digit were adequately removed thus creating a proximally based skin flap from the residual skin. Finally, the proximally based skin flap from the extra digit was brought to cover the medial wound defect (Fig. 3c and 3d).

Based on the above procedures, the modified technique seems to be more applicable particularly in selected patients with secondary type of congenital hallux varus. These patients tend to have excess skin from the extra digit medially which can be used for skin closure on the medial skin defect following the hallux varus reconstruction. This modified technique addresses more on the skin closure of the medial skin gap by using either proximally based or plantar-based skin flap derived from the residual skin of the extra digit rather than surgical release and fixation.

## Discussion

The primary difference in our technique compared to Farmer’s technique is on the skin cover, while the release of internal medial structures is similar. Farmer’s technique involves a risky process of rotating proximal based dorsal or plantar skin flap from the first web space to cover medial skin defect over the first metatarsophalangeal joint. In the modified procedure we used proximal based skin flap from the extra digit, thus avoiding rotational flap from the first web space. Suturing the proximally based skin flap from the extra digit to cover the defect could be done without tension, making it more likely to be successful. It is particularly suitable in the presence of polydactyly.

In the case of polysyndactyly, only a dorsal skin incision was made for clearance of bone and soft tissue of the extra digit as well as the release of medial metatarsophalangeal capsule and ligament to allow alignment of the big toe as in Fig. 2. It has a similar advantage as the proximally based flap as in the polydactyly.

In the Farmer technique, dissection of the skin on the first web space to raise the flap and to create a syndactyly carries a risk of compromising lateral circulation to the big toe, because of the close proximity of the neurovascular structures to the skin flap. In the modified technique, the decision of whether to remove the skin in the first web space or not depended on the width of the web space, which was not required in case No. 4.

The Farmer technique enhanced the stability of the big toe by creating a syndactyly following the K-wire transfixation of the big toe aligned to the first metatarsal. In our modified technique, the K-wire was essential to maintain the alignment of the great toe while awaiting the soft tissue to heal. During insertion, the K-wire might disturb the circulation to the tip of the big toe which was already compromised due to possible stretching of the medial and compression of the lateral digital vessels especially in the Farmer technique. We encountered this problem in case No. 1 in which we had to remove the K-wire and stabilise the toe with a cast temporarily. In cases No. 2 and No. 3, in which the modified technique was used, we were able to fix with K-wire without any circulatory compromise. We surmise that this was due to the better blood supply in the modified procedure.

This procedure required close monitoring of the big toe circulation after the surgery. If the circulation to the big toe was compromised, the K-wire may need to be removed, or syndactyly may need to be released, to relieve the tension to the neurovascular bundle. Therefore, during pre-operative counselling, parents had to be warned of the risk of ischemia, or worst, amputation of the big toe. There was also a risk of recurrence of the deformity.

Lengthening of the first metatarsal has been recommended in the presence of brachymetatarsia. We may consider future lengthening in situations similar to patients No. 2 and No. 4, which were not suitable to be performed during the first surgery because of doubtful vascularity. We also believe that distraction osteogenesis is not suitable for young children. who may not cooperate with the gradual turning of the distractor. In the case of a metaphyseal bracket, resection of the bracket followed by lengthening is recommended^3, 4, 5^.

The blood supply of the forefoot is derived from dorsum and plantar vessels. The forefoot is supplied by the dorsal metatarsal arteries and plantar metatarsal arteries which are branches from the arcuate artery and deep plantar arch, respectively. The medial side of the forefoot is also supplied by the superficial branch of the medial plantar artery. The big toe is supplied by the dorsal and plantar digital arteries which are branches from the dorsal and plantar metatarsal arteries on both medial and lateral aspects. In addition, the digital arteries on the medial aspect of the big toe also receive the blood supply directly from the medial plantar artery via its superficial branch.

Based on the knowledge of these vascular arrangements, we concluded that the technique of reconstruction can be modified according to the type and the deformity associated with the hallux varus. The modification with proximally or plantar-based flap can be a convenient and safe technique to close the skin defect.
